# High-Quality Draft Genome Sequences of Five *Xanthomonas arboricola* pv. fragariae Isolates

**DOI:** 10.1128/genomeA.01585-17

**Published:** 2018-02-15

**Authors:** Michael Gétaz, Steve Baeyen, Jochen Blom, Martine Maes, Bart Cottyn, Joël F. Pothier

**Affiliations:** a Environmental Genomics and Systems Biology Research Group, Institute for Natural Resource Sciences, Zurich University of Applied Sciences (ZHAW), Wädenswil, Switzerland; b Plant Sciences Unit—Plant Health, Flanders Research Institute for Agriculture, Fisheries and Food (ILVO), Merelbeke, Belgium; c Bioinformatics and Systems Biology, Justus-Liebig University Giessen, Giessen, Germany

## Abstract

*Xanthomonas arboricola* pv. fragariae was described in 2001 as the causal agent of strawberry bacterial leaf blight. We report here the first draft whole-genome sequences of five *X. arboricola* pv. fragariae isolates from Italy and France.

## GENOME ANNOUNCEMENT

*Xanthomonas arboricola* pv. fragariae was described in 2001 as the causal agent of strawberry bacterial leaf blight (1). The first symptoms related to this bacterium were observed in 1993 in strawberry cultivations in northern Italy (1). Afterward, the disease has only been reported in plantlets from Turkey in 2004 (2). The bacterium was under quarantine status in Europe starting in 2002 (3), but it was derestricted in 2007 (see https://www.eppo.int/MEETINGS/2007_meetings/phytomeasures.htm). This was partly due to the observation that the pathogen was sometimes coisolated with *Xanthomonas fragariae* (4, 5), which is the causal agent of bacterial angular leaf spot and is under quarantine status in Europe (6, 7). Also, the reported pathogenicity of *X. arboricola* pv. fragariae upon artificial inoculation on strawberry has often been ambiguous (4, 5, 8; M. Gétaz and J. F. Pothier, unpublished data). This could be related to the important heterogeneity among *X. arboricola* pv. fragariae strains, as observed in previous studies (4, 5, 9–11). Very recently, the pathogenicity of the pathotype strain was proved to produce typical symptoms in the strawberry cultivars Candonga, Sabrina, and Murano (12).

Until now, no whole-genome data for this bacterium were available in GenBank. In this study, whole-genome sequences of five *X. arboricola* pv. fragariae strains isolated from strawberry plants in Italy and France between 1986 and 1993 were obtained (Table 1).

**TABLE 1:** Draft whole-genome sequences of five *Xanthomonas arboricola* pv. fragariae strains submitted to the ENA database

Strain^a^	Synonym(s)	Yr, country of isolation	ENA accession no.	No. of contigs	Genome size (bp)	G+C content (%)	No. of CDSs
LMG 19145^b^	CFBP 6771, PD 2780	1993, Italy	OEQL00000000	39	4,906,785	65.88	4,019
LMG 19144	CFBP 6770, PD 2696	1993, Italy	OEQF00000000	58	4,842,182	65.94	3,954
CFBP 6762	PD 2694	1993, Italy	OEQE00000000	38	4,889,284	65.75	4,044
LMG 19146	CFBP 3548, PD 3164	1986, France	OEQG00000000	39	4,884,039	65.75	4,020
CFBP 6773	NA^c^	NA	OEQD00000000	39	4,692,498	65.95	3,852

a The culture collections providing strains are abbreviated in the strain names as LMG (Collection of the Laboratorium voor Microbiologie en Microbiele Genetica, Ghent, Belgium), CFBP (Collection Française de Bactéries Associées aux Plantes, Beaucouzé, France), or PD (Culture Collection of Plant Pathogenic Bacteria, Wageningen, the Netherlands).

b Pathotype strain.

c NA, not applicable.

Genomic DNA was extracted using the NucleoSpin tissue kit (Macherey-Nagel AG, Düren, Germany) following the manufacturer’s protocol. Paired-end libraries constructed by the Nextera XT DNA library prep kit (Illumina, San Diego, CA) were sequenced on a MiSeq system (Illumina) using a 600-cycle MiSeq reagent kit v3 (Illumina). *De novo* assemblies were created using SeqMan NGen from the Lasergene genomics package version 12.1.0 (DNAStar, Madison, WI). This was followed by contig reassembly using SeqMan Pro and read mapping using SeqMan NGen to check for inconsistencies.

The five genomes displayed an overall size between 4,692,498 and 4,906,785 bp, which is in the range previously observed with other *X. arboricola* genomes (13–17). The G+C contents in all the genomes were similar (65.75 to 65.95%). Genomes were annotated automatically using GenDB (18), and a total of 3,852 to 4,044 coding sequences (CDSs) were detected (Table 1). Using EDGAR 2.0 (19), a total of 3,523 CDSs were found to be shared between these five genomes.

Average nucleotide identities (ANIs) between these five genomes ranged from 96.57% to 97.69%, thus confirming the species designation but also suggesting some genome content heterogeneity.

The *X. arboricola* pv. fragariae draft genome sequences presented here add to the existing genomic information and further clarify the complexity of the species *X. arboricola*.

### Accession number(s).

The annotated draft whole-genome sequences of the five *X. arboricola* pv. fragariae isolates were deposited at ENA under the sequencing project number PRJEB23514. The accession numbers for the isolates are shown in Table 1.
